# Reinforcement Learning for Intraoperative Hypotension Management with Consideration to Postoperative Acute Kidney Injury

**DOI:** 10.34067/KID.0000001212

**Published:** 2026-05-05

**Authors:** Esra Adiyeke, Tianqi Liu, Venkata Sai Dheeraj Naganaboina, Han Li, Tyler J. Loftus, Yuanfang Ren, Benjamin Shickel, Matthew M. Ruppert, Karandeep Singh, Ruogu Fang, Parisa Rashidi, Azra Bihorac, Tezcan Ozrazgat-Baslanti

**Affiliations:** 1 Intelligent Clinical Care Center, University of Florida, Gainesville, Florida; 2 Division of Nephrology, Hypertension, and Renal Transplantation, Department of Medicine, University of Florida, Gainesville, Florida; 3 Department of Electrical and Computer Engineering, University of Florida, Gainesville, Florida; 4 Department of Computer Science, University of Florida, Gainesville, Florida; 5 Department of Surgery, University of Florida, Gainesville, Florida; 6 Department of Medicine, University of California San Diego, San Diego, California; 7 Department of Biomedical Engineering, University of Florida, Gainesville, Florida

**Keywords:** AKI, acute renal failure, BP, hypotension, vasopressin, artificial intelligence, cohort studies

## Abstract

**Key Points:**

- Intraoperative hypotension is associated with postoperative AKI, which is a common and morbid postoperative complication.
- We developed a reinforcement learning model to guide intraoperative fluids and vasopressors, avoiding hypotension and postoperative AKI.
- The model's policy showcases the potential to lower postoperative AKI and improve outcomes driven by intraoperative hypotension.

**Background:**

Traditional methods of surgical decision making heavily rely on human experience and prompt actions, which are variable. A data-driven system that generates treatment recommendations based on patient states can be a substantial asset in perioperative decision making for cases of intraoperative hypotension in which suboptimal management is associated with AKI, a common and morbid postoperative complication.

**Methods:**

In this retrospective cohort study, we analyzed 50,021 surgeries from 42,547 adult patients who underwent major surgery at a quaternary care hospital between 2014 and 2020. We developed a deep reinforcement learning model to recommend the optimum doses of intravenous fluids and vasopressors during surgery to avoid intraoperative hypotension and AKI defined by Kidney Disease Improving Global Outcomes serum creatinine criteria within 3 days after surgery.

**Results:**

The developed model replicated 69% of physicians' decisions for the dosage of vasopressors and proposed higher or lower dosage of vasopressors than received in 10% and 21% of the treatments, respectively. In terms of intravenous fluids, the model's recommendations were within 0.05 ml/kg per 15 minutes of the actual dose in 41% of the cases, with higher or lower doses recommended for 27% and 32% of the treatments, respectively. The reinforcement learning policy resulted in a higher estimated policy value compared with the physicians' actual treatments, as well as random policies and zero-drug policies. AKI incidence was the lowest in patients who received medication dosages that aligned with our agent model's decisions.

**Conclusions:**

Our findings suggest that implementation of the model's policy has the potential to lower postoperative AKI and improve other outcomes driven by intraoperative hypotension.

## Visual Abstract

**Figure:**
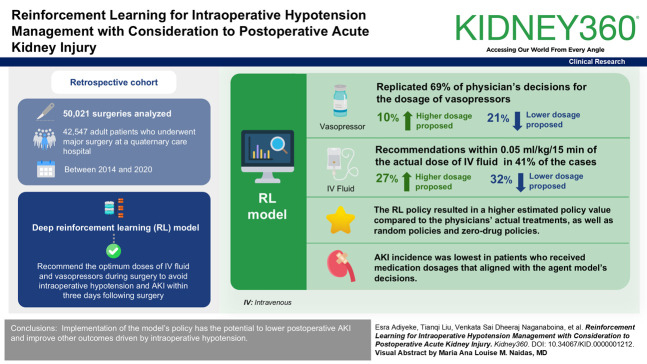

## Introduction

Postoperative AKI affects almost 20% of the patients undergoing surgery and poses substantial risk for short- and long-term organ dysfunction and mortality.^1–7^ Management of intravascular volume and vasomotor tone plays a substantial role in determining the risk of postoperative AKI.^6,8,9^ Accordingly, previous research has shown associations between intraoperative hypotension and AKI.^10,11^ Despite this importance, there are few consensus guidelines regarding best practices for predicting and treating intraoperative hypotension.^12^ The Acute Disease Quality Initiative and Perioperative Quality Initiative recommended maintaining mean arterial pressure >65 mm Hg based on moderate quality evidence, and recommended goal-directed BP optimization based on stronger evidence.^5,13,14^ Two randomized trials have demonstrated that goal-directed therapy lowers the odds of postoperative AKI and another demonstrated that machine learning-derived predictions of impending hypotension prompt anesthesiologists to act earlier, differently, and more frequently in managing intravascular volume and vasomotor tone, resulting in less time-weighted hypotension.^13,15^

Reinforcement learning is an artificial intelligence subfield that identifies the sequence of actions yielding the greatest probability of achieving a goal, such as normal postoperative renal function.^16^ Previous works have applied reinforcement learning algorithms to situations requiring real-time decisions accounting for a patient's continuously changing states,^17–19^ including sepsis,^20–23^ sedation,^24^ pain management,^25^ diabetic glycemic management,^26^ and delirium.^27^ Reinforcement learning has been particularly studied in sepsis management, where agents have been refined to account for model uncertainty,^28^ multidimensional drivers of sepsis mortality,^29^ and physician input to optimize fluid and vasopressor dosages.^30^ In hypotension management, Futoma *et al*.^31^ developed a reinforcement learning approach to suggest multiple equivalent actions in an intensive care unit (ICU) setting. Zhang *et al*.^28^ further refined reinforcement learning optimization of ICU hypotension treatment by limiting an agent to only suggest actions at critical decision-making points, rather than at time intervals throughout the length of an ICU stay. However, there are currently no reinforcement learning agents to minimize hypotension-related complications in the perioperative setting.

Our objective is to develop and validate a deep *Q* networks-based reinforcement learning model that offers optimal dosing strategies for intravenous fluid and vasopressor administration for every 15-minute interval during a major surgery to maintain optimal hemodynamic physiology and avoid postoperative AKI.

## Methods

### Data Source

The University of Florida Integrated Data Repository was the honest broker to assemble a single-center, longitudinal perioperative cohort for all patients admitted to the University of Florida Health for patients of age 18 years or older during admission, following any type of major operative procedure between June 1, 2014, through September 20, 2020, by integrating electronic health records data from preoperative, intraoperative, and postoperative phases of care with other clinical, administrative, and public databases, as previously described.^32^ We excluded patients with ESKD, who underwent cardiac surgery, died within 24 hours of surgery, had insufficient data, and patients with hospital stays <24 hours. The final cohort consisted of 50,021 inpatient encounters who underwent major surgery (Supplemental Figure 1). We chronologically split the cohort into development (admissions between June 1, 2014, and November 29, 2018, 70% of the entire cohort) and test (admissions between November 30, 2018, and September 20, 2020, 30% of the entire cohort) sets. We trained the model using the development cohort, allocated 30% for internal validation and parameter tuning, and reported model performance on the test set. If patients underwent multiple surgeries during an admission, only the first surgery was considered in the analyses. As some patients had multiple surgical encounters, we restricted the analysis to the first eligible major surgery per surgical encounter. The University of Florida Institutional Review Board and Privacy Office approved this study with waiver of informed consent (IRB#201600223, IRB#201600262). Our study adhered to the Transparent Reporting of a multivariable prediction model for Individual Prognosis or Diagnosis guidelines.^33^

### Assessment of Kidney Function

In determining AKI and CKD, we considered Kidney Disease Improving Global Outcomes serum creatinine criteria.^34^ We considered preadmission and admission serum creatinine records in determining baseline creatinine. We computed creatinine by back-calculation using the 2021 CKD Epidemiology Collaboration refit without race equation assuming a baseline eGFR of 75 ml/min per 1.73 m^2^ in cases with no preadmission creatinine was available in non-CKD patients.^35^ We refer to a study by Ozrazgat-Baslanti *et al*. for all necessary details regarding the data elements, assumptions, and algorithms of the computable phenotyping pipeline we developed and used.^36^

### Predictor Features

The model development used 17 features, four static baseline features (age, sex, Charlson Comorbidity Index, body mass index), nine intraoperative physiologic time series data (heart rate, systolic BP, mean arterial pressure, body temperature, respiratory rate, minimum alveolar concentration, saturation of peripheral blood oxygen saturation/fraction of inspired oxygen ratio, peak inspiratory pressure, and end-tidal carbon dioxide), one preoperative feature (the average mean arterial BP of past 48 hours), and two additional features (cumulative intravenous fluid and cumulative pressor administered during surgery). We derived preoperative comorbidities from International Classification of Diseases codes to calculate Charlson comorbidity indices.^37^ We computed the mean values of vital signs from intraoperative time series data by resampling into 15-minute intervals. List of all input features and their statistical characteristics is given in Supplemental Tables 1 and 2. We reported the differences between groups using the independent *t* test or Mann–Whitney *U* test for continuous variables and the Chi-square or Fisher exact test for discrete variables as appropriate.

### Reinforcement Learning Model

**Figure 1:**
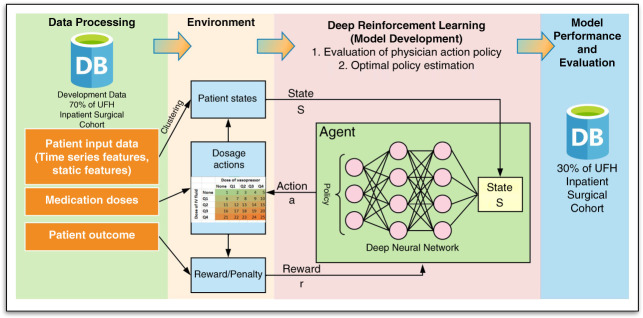
**Data flow and design of reinforcement learning approach using deep learning framework.** Data from our adult surgical cohort included static and times series features. We defined the action space with a total of 25 discrete actions, combining five different levels of intravenous fluid and vasopressor dosages. The model environment consists of electronic health records information, reward function, and associated actions. Environment supplies the observable states to the agent. Agent analyzes the state using a deep neural network trained to produce the best action given optimum policy. The suggested action is sent to the environment, and the scored reward due to the action is sent back to the agent for training the policy. Model was trained and validated on 70% of the data and tested on 30% of the data, respectively. UFH, University of Florida Health.

The proposed reinforcement learning algorithm is conceptualized as a dynamic model that uses preoperative and intraoperative features to recommend actions during surgery. We modeled for action prediction to prevent short-term outcome of intraoperative hypotension (mean arterial pressure <65 mm Hg) and long-term outcome of AKI in the first 3 days after the surgery.^10^ The resulting actions (agent policy) are assessed compared with the actions taken by the physicians on the basis of their experience (Figure 1). This workflow simulates clinical tasks faced by physicians involved in perioperative care where patients' preoperative information is subsequently enriched by the influx of new data from the operating room. The final output produces the suggested actions in a dynamic manner, updating the suggestions every 15 minutes.

The algorithm consists of two main layers: data processing and modeling. Each contains a data transformer core and a data analytics core.^32^ Briefly, the workflow uses a data transformer to integrate preoperative data with the data from surgery rooms and prepares the data for analysis through preprocessing and feature transformation (Supplemental Figure 2). We used Dueling Double Deep *Q* Networks as architecture for the reinforcement learning model to test and analyze the optimal behavior. We discretized the state space by clustering the clinical variables which were resampled at 15-minute intervals as described in the Predictor Features section. Using the K-means++ clustering algorithm, we grouped patients to capture high-level similarities and then computed transition probabilities by considering the transition counts. We determined the number of clusters using silhouette analysis and selected 200 as the optimal value (Supplemental Figure 3). We developed 50 distinct reinforcement learning models for 50 different clustering outcomes obtained by different random initializations to account for the variability in policy values. We considered SHapley Additive exPlanations to evaluate the features' influences in reinforcement learning model.^38^ We used an enhanced version of the Deep Learning Important FeaTures algorithm to approximate the SHapley Additive exPlanations values.^39^ Deep Learning Important FeaTures can decompose the prediction of a neural network on a specific input by backpropagating the contributions of all neurons in the network to every feature of the input. We set the model running for 300 epochs, the batch size of 256 experiences, and a learning rate of 1e-3.

### Action Space and Reward Function

We converted the vasopressors using their NE equivalent in mcg/kg per minute (Supplemental Table 3).^40^ We discretized the action space into five groups using cutoff values derived from the empirical distribution of historical actions to ensure sufficient representation for each action category for intravenous fluids and vasopressors separately, one group corresponding to dosage of zero (Supplemental Tables 4 and 5). We defined the action space with a total of 25 discrete actions, combining five different levels of intravenous fluid and five different levels of vasopressor dosages. We developed a reward function as a combination of long- and short-term rewards with two major parts to consider. In modeling the postoperative outcomes, we assigned a long-term reward (or penalty) using a dedicated reward of +15 if no AKI occurs within 3 days following the surgery and −15 otherwise. The second part of the reward function adds a penalty of −1.75 if the state is hypotensive; that is, if mean arterial pressure is <65 mm Hg or 20% lower than baseline mean arterial pressure. We calculated the baseline mean arterial pressure by calculating the median noninvasive mean arterial pressure within the 48 hours before the first surgery start date-time that was between 60 and 110 mm Hg. Additionally, we explored incorporating an alternative stability-based reward to encourage sustained hemodynamic control where mean arterial pressure in the normal range earned +3, deviation from normal range −3, and sudden means arterial pressure changes defined as change >20% were penalized by −3. For the long-term reward (penalty), we evaluated a version in which the reward value of +15 (or a penalty value of −15) was applied only at the end of the surgery. We explored an alternative reward function and reported the outcomes in Supplemental Figure 4. This reward version resulted in action distribution less similar to physicians' actions and higher return values corresponding to higher AKI incidence. Thus, we continued our analyses with version described above.

### Performance Evaluation

In reinforcement learning, model performance is typically evaluated through interaction with a simulated environment using metrics like cumulative reward. However, because of lack of such environment in the current scenario, we assessed the model's policy quality through action prediction accuracy and off-policy evaluation techniques. To study that, we adopted visual analysis and weighted importance sampling as the quantitative method.^20,41,42^ We adopted an off-policy evaluation to quantitatively assess our trained agent ($\pi_{e}$) based on physicians' policies. Weighted importance sampling takes the idea to reweight the rewards in the historical data (physicians' policy $\pi_{b}$) by the importance sampling ratio between $\pi_{e}$ and $\pi_{b}$. For visual analysis, policy scores of the model were compared with the physician policy relating the scores to outcome prevalence, along with distribution of actions in action space. We also evaluated the impact of difference in actions on the long-term outcome prevalence. To address the instability of evaluation performance in long-term cases, we propose a modified version of weighted importance sampling for long-term applications. To clarify, we used logarithmic transformation combined with Softmax to constrain the range of the cumulative importance ratio, improving stability in long-term experiments (Supplemental Methods).

## Results

### Patient Baseline Characteristics and Outcomes

Among the 28,586 patients with 34,186 major surgeries in the development cohort, the mean age was 57 (SD, 17), 17,031 (50%) were female, 4776 (14%) were Black, 1530 (4%) were Hispanic, and 15,578 (46%) had insurance through Medicare (Table 1 and Supplemental Table 2). The test cohort consisted of 13,961 patients with 15,835 major surgeries. In this cohort, the mean age was 59 (SD, 17), 7909 (50%) were female, 2233 (14%) were Black, 756 (5%) were Hispanic, and 7672 (48%) had insurance through Medicare. The most common types of surgery, in descending order of frequency, were orthopedic, neurosurgical, and vascular procedures in both cohorts. The prevalence of postoperative complications in the development cohort was 11% for AKI within the first 3 days following surgery, 2% for 30-day mortality, and 4% for 90-day mortality. In the test cohort, the percentage was 12% for postoperative AKI, 2% for 30-day mortality, and 3% for 90-day mortality. AKI occurred more frequently in patients with intraoperative hypotension than in those without (12% versus 10%, *P* < 0.001; Supplemental Table 6).

**Table 1:** Clinical characteristics and outcomes of the patients

Feature	Development Cohort	Test Cohort
Number of encounters, *No.*	34,186	15,835
**Demographic information**		
Age, yr, mean (SD)	57 (17)	59 (17)
Female, *No.* (%)	17,155 (50)	7926 (50)
Black, *No.* (%)	4776 (14)	2233 (14)
BMI, median (IQR)	28 (24–34)	28 (24–34)
Emergency admission, *No.* (%)	12,243 (36)	5763 (36)
**Baseline clinical information**		
Three most common types of surgery, *No.* (%)		
*Orthopedic surgery*	10,109 (30)	4341 (27)
*Neurosurgery*	3744 (11)	2546 (16)
*Vascular surgery*	3420 (10)	1704 (11)
Charlson Comorbidity Index, median (IQR)	4 (2–6)	4 (2–6)
Reference eGFR (ml/min per 1.73 m^2^), median (IQR)	95 (82–110)	93 (81–108)
Baseline mean arterial pressure mm Hg, median (IQR)^a^	86 (78–94)	87 (79–95)
**Intraoperative vitals, median (IQR)**		
Systolic BP, mm Hg	114 (102–130)	116 (104–132)
Mean arterial pressure, mm Hg	79 (70–90)	82 (72–93)
Heart rate, beats/min	75 (66–87)	75 (66–87)
SpO_2_, %	99 (98–100)	99 (97–100)
FiO_2_, %	40 (40–40)	40 (40–40)
EtCO_2_, mm Hg	34 (32–37)	35 (33–38)
Respiration rate, breaths/min	10 (8–12)	12 (10–14)
Peak inspiratory pressure, mm Hg	18 (14–23)	18 (14–22)
MAC	0.6 (0.4–0.8)	0.6 (0.3–0.8)
Core temperature, degrees Celsius	36.8 (36.3–37.3)	36.9 (36.3–37.4)
**Intraoperative medications, median (IQR)** ^b^		
Vasopressor total dose per 15 min (mcg/kg)	0 (0–0)	0 (0–0.1)
Intravenous fluids total dose per 15 min (ml/kg)	0.1 (0–0.4)	0.1 (0–0.4)
**Intraoperative hypotension, *No.* (%)**	24,144 (70)	9677 (61)
Postoperative AKI, *No.* (%)		
AKI within 3 d after surgery	3870 (11)	1856 (12)
AKI within 7 d after surgery	4637 (14)	2242 (14)
AKI during hospitalization	5649 (17)	27,72 (18)
**Mortality, *No.* (%)**		
30-d mortality	749 (2)	320 (2)
90-d mortality	1350 (4)	475 (3)

BMI, body mass index; EtCO_2_, end-tidal carbon dioxide; FiO_2_, fraction of inspired oxygen; ICU, intensive care unit; IQR, interquartile range; MAC, minimum alveolar concentration; SpO_2_, oxygen saturation.

a Baseline mean arterial pressure was calculated as the median noninvasive mean arterial pressure within 48 hours before the start of the first surgery, limited to values between 60 and 110 mm Hg.

b Values were calculated for 15-minute resampled series.

### Model Evaluation

**Figure 2:**
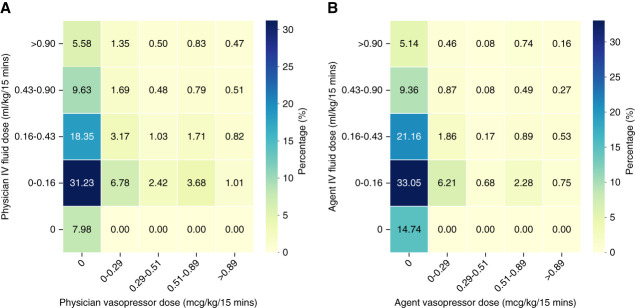
**Action distributions of the physicians and trained agent.** Comparison of actions taken by physician agent (A) and proposed by the trained agent (B). Each bin represents the tuple for discretized intravenous fluids and vasopressor actions. IV, intravenous.

**Figure 3:**
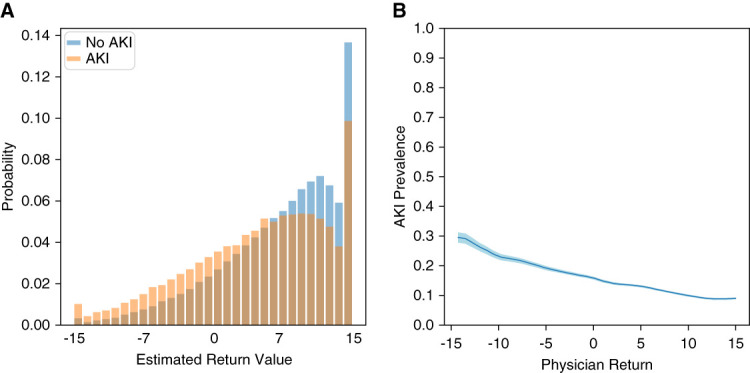
**Average return per surgery and the relationship between the return of physicians' treatments and postoperative AKI.** (A) Distribution of average returns for cases with and without postoperative AKI. (B) Relationship between physicians' return values and AKI prevalence in the test set.

**Figure 4:**
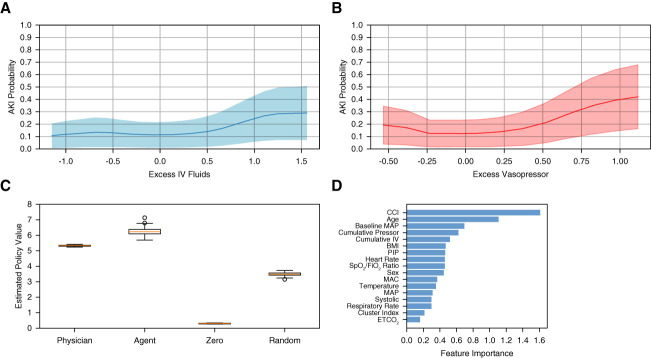
**Average dose excess outcomes, estimated value distributions and feature importance.** The average dose excess, calculated as the difference between given and recommended dose over all 15-minute intervals for intravenous fluids (A) and for vasopressors (B) per surgery. (C) Comparison of estimated policy value by weighted importance sampling for physician policy, agent policy, zero policy, and random policy with 50 different K-Means initializations. (D) Feature importance obtained for reinforcement learning model. BMI, body mass index; CCI, Charlson Comorbidity Index; EtCO_2_, end-tidal carbon dioxide; MAC, minimum alveolar concentration; MAP, mean arterial pressure; PIP, peak inspiratory pressure; SpO_2_, oxygen saturation.

We trained the Dueling Double Deep *Q* Networks-based reinforcement learning models on a development cohort of 34,186 surgeries, resampled to 545,965 distinct 15-minute epochs. All results were reported from a test cohort of 15,835 surgeries, resampled to 255,748 distinct 15-minute epochs. We illustrated different aspects of agent suggestion evaluation in Figures 2–4. Action space distribution in Figure 2 suggests that actions recommended by the agent and actions taken by the physicians are similar. The average *Q*-value distributions for surgeries that were grouped by the presence or absence of postoperative AKI are illustrated in Figure 3A. We observed that surgeries with postoperative AKI tended to have lower return values, and in contrast, surgical sessions without AKI concluded in higher return values. Figure 3B presents the relationship between physicians' actions and postoperative AKI within the first 3 days after surgery. In Figure 3B, physicians' treatments with a higher return value correspond to lower AKI probability, whereas treatments with a low return led to higher AKI probability.

On average, the reinforcement learning model recommends less vasopressors and less intravenous fluids. The model replicated the physicians' vasopressor dosing decisions in 69% of cases, and it recommended a higher dose in 10% of treatments and a lower dose in 21% of treatments. For intravenous fluids, the actual dose was within 0.05 ml/kg per 15 minutes of the model's suggestion in 41% of cases; in the remainder, the model's recommendation was higher in 27% and lower in 32% of treatments. Figure 4 demonstrates that administering either treatment at doses higher or lower than those recommended by the artificial intelligence policy was associated with a higher probability of AKI. We presented the distribution of the estimated policy value of the physicians' actual treatments, the reinforcement learning policy, a random policy, and a zero-drug policy in Figure 4C, where estimated policy value is the expected cumulative reward each policy would yield over time. In this figure, the reinforcement learning policy resulted in a higher estimated value compared with the alternatives. The order for the most influential variables in the reinforcement learning model was given Figure 4D. We identified Charlson Comorbidity Index and age as the top two important features in the decision-making process.

### Example Surgeries

We illustrated four example surgical sessions including cases with and without postoperative AKI within the first 3 days after surgery (Supplemental Figures 5–8). In example patients presented in Supplemental Figures 5 and 6, they did not experience hypotensive episodes and did not have postoperative AKI. We observed the physician tended to administer intravenous fluids more and vasopressors almost each hour (when mean arterial pressure fluctuated around 65 mm Hg) for patients represented in Supplemental Figure 5 compared with the administered intravenous fluids for patient with the 15-minute average mean arterial pressure remained consistently above 90 mm Hg (Supplemental Figure 6). In both cases, the reinforcement learning model did not recommend vasopressor administration. Similarly, the physician administered more intravenous fluids in the former case compared with the reinforcement learning model, while the model recommended more fluids in the latter case. In the hypotensive example with postoperative AKI given in Supplemental Figure 7, the reinforcement learning model recommends higher amounts of intravenous fluids from the start and maintains this level throughout most of the surgery compared to the physician's administration. Additionally, the model recommends the use of vasopressors, whereas the physician chose not to administer them. In the case of a nonhypotensive surgical patient who had postoperative AKI, the model's recommendations aligned with the physicians' decision to not administer vasopressors administration and to administer similar intravenous fluid on average (Supplemental Figure 8).

## Discussion

Postoperative AKI is prevalent and occurs in almost one in five patients following a major surgery.^7^ Intraoperative hypotension affects approximately one in three patients undergoing noncardiac surgical interventions.^43^ Prior research has demonstrated strong associations between intraoperative hypotension and postoperative AKI.^44^ Improving the management of intraoperative hypotension during noncardiac surgery could save hospitals up to $4.6 million annually, primarily by decreasing the incidence of postoperative AKI.^45^

In this study, we introduced a reinforcement learning modeling approach aimed at assisting physicians in preventing intraoperative hypotension and reducing the risk of postoperative AKI by providing optimal intravenous fluid and vasopressor dosage recommendations. Traditional machine learning methods primarily focus on outcome prediction or risk stratification using historical data and do not explicitly model the effects of clinical actions on future patient states. In contrast, reinforcement learning models are designed to learn decision policies that map patient states to recommended actions while optimizing cumulative benefit over time. This policy-centric framework enables reinforcement learning approach to support sequential decision making, where interventions influence downstream outcomes. Furthermore, this approach is suited for complex clinical scenarios in which no universally agreed-upon ground truth or optimal treatment pathway exists, as it learns from observed outcomes rather than predefined labels. The model demonstrated promise in recommending actions that are associated with improved outcomes. Although the actions recommended by the agent and the actions taken by physicians had a high degree of similarity, administering higher doses of either intravenous fluids or vasopressors than those recommended by the reinforcement learning policy was associated with a higher probability of postoperative AKI; similarly, administering lower doses of vasopressors than recommended by the artificial intelligence policy was also (although weakly) associated with a higher probability of postoperative AKI. Noting that, in this proposed framework, the model was trained to maximize expected return based on reward function that penalizes both intraoperative hypotension and postoperative AKI. On average, the model recommends less vasopressor or intravenous fluids than physician actions. This behavior may be due to model being aware of the cumulative vasopressor and intravenous fluids administered at each time step during the surgery as they were provided as input variables to the model, to prevent dangers of fluid overload and excessive vasopressor. This may suggest that the model may be capturing the trade-off between avoiding hypotension and avoiding treatment-related adverse effects.

We showed that the reinforcement learning policy resulted in a higher estimated value compared with the alternatives, including treatments administered by physicians. Previous studies dedicated to this issue rely heavily on the correctness of physician actions, which may potentially replicate nonevidence-based practices. One possible solution could involve the comparison of physician actions to actions recommended by multiple subject matter experts in various scenarios, especially in scenarios where patients experienced better or worse than expected outcomes. There exists no optimal, one-size-fits-all approach to clinical decision making.^17,46^

Although our results show promise, there are numerous challenges associated with implementing such a method in health care, especially in perioperative settings, where reinforcement learning performance cannot be easily quantified. Unlike simulated synthetic environments, clinical settings do not permit real-time experimentation with unvalidated policies. Thorough model validation is required to ensure patient safety. Current literature describing the application of reinforcement learning in health care focuses on its usage in dynamic environments to optimize treatment regimens. These dynamic conditions, such as sepsis, require that current models adapt rapidly to evolving patient states to avoid decompensation and physiological failure. Thus, one challenge to reinforcement learning algorithms is the frequency of treatment recommendations. In their model for sepsis, another condition partially defined by hypotension, Komorowski *et al*.^20^ and Tang *et al*.^30^ aggregated patient data every 4 hours for sepsis treatment. The shortest timestep used in current literature was 1 hour.^21,24,31^ Zhang *et al*.^47^ used a model that specifically identified highly variable decision-making points among physicians to make treatment recommendations. Yu *et al*.^24^ used 10-minute intervals to accomplish optimal mechanical ventilation, a treatment that is similar to fluid resuscitation in its timing and necessity. Peine *et al*.^48^ used 4-hour time steps to dynamically optimize mechanical ventilation regime for critically ill patients. Prasad *et al*.^49^ used a 10-minute timestep, but they optimize mechanical ventilation and sedation dosage and weaning from mechanical ventilation. In this work, we chose a 15-minute time interval under the assumption that intraoperative hypotension requires rapid correction, especially in a perioperative environment.

Future work may explore the integration of multimodal intraoperative data to improve representation of patient state, while recognizing current limitations in real-time availability and feasibility. As access to such data modalities improves, multimodal approaches may support more patient-specific and context-aware recommendations.^50^

We present a reinforcement learning modeling approach to recommend optimal intravenous fluid and vasopressor doses to avoid intraoperative hypotension and postoperative AKI. AKI incidence was lowest when clinician actions closely aligned with the model's recommendations. These findings require prospective validation and clinical implementation to assess the potential to improve perioperative care and patient outcomes.

## Supplementary Material

## Data Availability

Original data generated for the study will be made upon reasonable request to the corresponding author. Data Type: Observational Data; Health Care Data. Reason for Restricted Access: Data used in this analysis include both date and time stamps. In order to prevent patient privacy compromises due to inclusion of identifiers in the data, our data cannot be publicly shared in a repository. Author contact is Azra Bihorac (abihorac@ufl.edu).

## Declarative Statements

This study received Institutional Review Board or Ethics Committee approval. The need to obtain informed patient consent was waived. This research was posted on a preprint server. https://arxiv.org/abs/2505.21596.

## Supplemental Material

Supplemental Methods.

Supplemental Figure 1. Figure illustrating derivation of the study population.

Supplemental Figure 2. Workflow pipeline.

Supplemental Figure 3. The silhouette score (A) and within-cluster sum of squares (B) as functions of the number of clusters.

Supplemental Figure 4. Comparison of actions proposed by the trained agent (A) and taken by physician agent (B), average return per surgery (C) and the relationship between the return of physicians' treatments and postoperative AKI within the first 3 days following surgery in test set (D), difference between given and recommended dose over all 15-minute intervals for intravenous fluids (E), and for vasopressors (F) per surgery.

Supplemental Figure 5. Comparison between doses suggested by reinforcement learning model and physicians' administration for a surgical session without postoperative AKI within the first 3 days after surgery. (Example surgery 1).

Supplemental Figure 6. Comparison between doses suggested by reinforcement learning model and physicians' administration for a surgical session without postoperative AKI within the first 3 days after surgery. (Example surgery 2).

Supplemental Figure 7. Comparison between doses suggested by reinforcement learning model and physicians' administration for a surgical session with postoperative AKI within the first 3 days after surgery. (Example surgery 3).

Supplemental Figure 8. Comparison between doses suggested by reinforcement learning model and physicians' administration for a surgical session with postoperative AKI within the first 3 days after surgery. (Example surgery 4).

Supplemental Table 1. Table listing characteristics of input variables.

Supplemental Table 2. Detailed cohort characteristics.

Supplemental Table 3. NE conversion factors.

Supplemental Table 4. Intravenous fluid discretization.

Supplemental Table 5. Vasopressor discretization.

Supplemental Table 6. Hypotension and AKI outcomes.
