# Supplementary material for: Reinforcement Learning for Intraoperative Hypotension Management with Consideration to Postoperative Acute Kidney Injury
Source: Kidney360. 2026 May 5;7(7):1535–44. doi: 10.34067/KID.0000001212 (PMC13450969; doi:10.34067/KID.0000001212)
Supplement: Supplementary file 1 [file kidney360-7-1535-s001.pdf]

ASN American Society of Nephrology | JASN Journal of the American Society of Nephrology | KIDNEY360 Accessing Our World From Every Angle | CJASN Clinical Journal of the American Society of Nephrology

ASN Journal Disclosure Form

As per ASN journal policy, I have disclosed any financial relationships or commitments I have held in the past 36 months as included below. I have listed my Current Employer below to indicate there is a relationship requiring disclosure. If no relationship exists, my Current Employer is not listed.

E. Adiyeke reports the following:
Employer: University of Florida

I understand that the information above will be published within the journal article, if accepted, and that failure to comply and/or to accurately and completely report the potential financial conflicts of interest could lead to the following: 1) Prior to publication, article rejection, or 2) Post-publication, sanctions ranging from, but not limited to, issuing a correction, reporting the inaccurate information to the authors' institution, banning authors from submitting work to ASN journals for varying lengths of time, and/or retraction of the published work.

Name: Esra Adiyeke
Manuscript ID: K360-2025-001097R1
Manuscript Title: Reinforcement learning for intraoperative hypotension management with consideration to postoperative acute kidney injury
Date of Completion: February 18, 2026
Disclosure Updated Date: August 11, 2025

ASN American Society of Nephrology
JASN Journal of the American Society of Nephrology
KIDNEY360 Accessing Our World From Every Angle
CJASN Clinical Journal of the American Society of Nephrology

ASN Journal Disclosure Form

As per ASN journal policy, I have disclosed any financial relationships or commitments I have held in the past 36 months as included below. I have listed my Current Employer below to indicate there is a relationship requiring disclosure. If no relationship exists, my Current Employer is not listed.

A. Bihorac reports the following:
Employer: University of Florida; Research Funding: NIH; Current Grants: R25 GM155478; R01 GM110240; OT2 OD032701; NEPHROCLEAR CCL14 Test; GM149657; DK129541; DK121730-01A1; NS120924-01; and Patents or Royalties: Method and Apparatus for Prediction of Complications After Surgery. US Patent Number 20200161000. Filed 6/1/2018; Published 5/21/2020;; Systems and Methods for Providing an Acuity Score for Critically Ill or Injured Patients. US Patent Number WO2020172607A12020. Filed 2/21/2020. Published 8/27/2020. Active 6/24/2025. US12340905B2;; Method and apparatus for pervasive patient monitoring. US Patent Number 20190326013. Filed 4/18/2019; Published 10/24/2019.

I understand that the information above will be published within the journal article, if accepted, and that failure to comply and/or to accurately and completely report the potential financial conflicts of interest could lead to the following: 1) Prior to publication, article rejection, or 2) Post-publication, sanctions ranging from, but not limited to, issuing a correction, reporting the inaccurate information to the authors' institution, banning authors from submitting work to ASN journals for varying lengths of time, and/or retraction of the published work.

Name: Azra Bihorac
Manuscript ID: K360-2025-001097R2
Manuscript Title: Reinforcement learning for intraoperative hypotension management with consideration to postoperative acute kidney injury
Date of Completion: April 6, 2026
Disclosure Updated Date: April 6, 2026

ASN American Society of Nephrology | JASN Journal of the American Society of Nephrology | KIDNEY360 Accessing Our World From Every Angle | CJASN Clinical Journal of the American Society of Nephrology

ASN Journal Disclosure Form

As per ASN journal policy, I have disclosed any financial relationships or commitments I have held in the past 36 months as included below. I have listed my Current Employer below to indicate there is a relationship requiring disclosure. If no relationship exists, my Current Employer is not listed.

R. Fang reports the following:
Employer: University of Florida

I understand that the information above will be published within the journal article, if accepted, and that failure to comply and/or to accurately and completely report the potential financial conflicts of interest could lead to the following: 1) Prior to publication, article rejection, or 2) Post-publication, sanctions ranging from, but not limited to, issuing a correction, reporting the inaccurate information to the authors' institution, banning authors from submitting work to ASN journals for varying lengths of time, and/or retraction of the published work.

Name: Ruogu Fang
Manuscript ID: K360-2025-001097R1
Manuscript Title: Reinforcement learning for intraoperative hypotension management with consideration to postoperative acute kidney injury
Date of Completion: February 23, 2026
Disclosure Updated Date: February 23, 2026

ASN American Society of Nephrology | JASN Journal of the American Society of Nephrology | KIDNEY360 Accessing Our World From Every Angle | CJASN Clinical Journal of the American Society of Nephrology

ASN Journal Disclosure Form

As per ASN journal policy, I have disclosed any financial relationships or commitments I have held in the past 36 months as included below. I have listed my Current Employer below to indicate there is a relationship requiring disclosure. If no relationship exists, my Current Employer is not listed.

H. Li has nothing to disclose.

I understand that the information above will be published within the journal article, if accepted, and that failure to comply and/or to accurately and completely report the potential financial conflicts of interest could lead to the following: 1) Prior to publication, article rejection, or 2) Post-publication, sanctions ranging from, but not limited to, issuing a correction, reporting the inaccurate information to the authors' institution, banning authors from submitting work to ASN journals for varying lengths of time, and/or retraction of the published work.

Name: Han Li
Manuscript ID: K360-2025-001097R2
Manuscript Title: Reinforcement learning for intraoperative hypotension management with consideration to postoperative acute kidney injury
Date of Completion: April 8, 2026
Disclosure Updated Date: March 25, 2026

ASN American Society of Nephrology | JASN Journal of the American Society of Nephrology | KIDNEY360 Accessing Our World From Every Angle | CJASN Clinical Journal of the American Society of Nephrology

ASN Journal Disclosure Form

As per ASN journal policy, I have disclosed any financial relationships or commitments I have held in the past 36 months as included below. I have listed my Current Employer below to indicate there is a relationship requiring disclosure. If no relationship exists, my Current Employer is not listed.

T. Liu reports the following:
Employer: Tencent America,; and Ownership Interest: Google, Meta, Nvidia, AMD, Tencent,.

I understand that the information above will be published within the journal article, if accepted, and that failure to comply and/or to accurately and completely report the potential financial conflicts of interest could lead to the following: 1) Prior to publication, article rejection, or 2) Post-publication, sanctions ranging from, but not limited to, issuing a correction, reporting the inaccurate information to the authors' institution, banning authors from submitting work to ASN journals for varying lengths of time, and/or retraction of the published work.

Name: Tianqi Liu
Manuscript ID: K360-2025-001097R1
Manuscript Title: Reinforcement learning for intraoperative hypotension management with consideration to postoperative acute kidney injury
Date of Completion: February 19, 2026
Disclosure Updated Date: February 19, 2026

ASN
American Society of Nephrology
JASN
Journal of the American Society of Nephrology
KIDNEY360
Accessing Our World From Every Angle
CJASN
Clinical Journal of the American Society of Nephrology

ASN Journal Disclosure Form

As per ASN journal policy, I have disclosed any financial relationships or commitments I have held in the past 36 months as included below. I have listed my Current Employer below to indicate there is a relationship requiring disclosure. If no relationship exists, my Current Employer is not listed.

T. Loftus reports the following:
Employer: University of Florida Health; Patents or Royalties: I am a named inventor on a patent that is not associated with a for-profit company.; and Advisory or Leadership Role: Unpaid membership on the Board of Directors for a non-profit educational collaborative.

I understand that the information above will be published within the journal article, if accepted, and that failure to comply and/or to accurately and completely report the potential financial conflicts of interest could lead to the following: 1) Prior to publication, article rejection, or 2) Post-publication, sanctions ranging from, but not limited to, issuing a correction, reporting the inaccurate information to the authors' institution, banning authors from submitting work to ASN journals for varying lengths of time, and/or retraction of the published work.

Name: Tyler J. Loftus
Manuscript ID: K360-2025-001097R1
Manuscript Title: Reinforcement learning for intraoperative hypotension management with consideration to postoperative acute kidney injury
Date of Completion: February 18, 2026
Disclosure Updated Date: February 18, 2026

ASN Journal Disclosure Form

As per ASN journal policy, I have disclosed any financial relationships or commitments I have held in the past 36 months as included below. I have listed my Current Employer below to indicate there is a relationship requiring disclosure. If no relationship exists, my Current Employer is not listed.

V. Naganaboina reports the following:
Employer: University of Florida; Amazon LLC.; and Ownership Interest: Amazon LLC.

I understand that the information above will be published within the journal article, if accepted, and that failure to comply and/or to accurately and completely report the potential financial conflicts of interest could lead to the following: 1) Prior to publication, article rejection, or 2) Post-publication, sanctions ranging from, but not limited to, issuing a correction, reporting the inaccurate information to the authors' institution, banning authors from submitting work to ASN journals for varying lengths of time, and/or retraction of the published work.

Name: Venkata Sai Dheeraj Naganaboina
Manuscript ID: K360-2025-001097R1
Manuscript Title: Reinforcement learning for intraoperative hypotension management with consideration to postoperative acute kidney injury,
Date of Completion: February 24, 2026
Disclosure Updated Date: February 24, 2026

ASN American Society of Nephrology · JASN Journal of the American Society of Nephrology · KIDNEY360 Accessing Our World From Every Angle · CJASN Clinical Journal of the American Society of Nephrology

ASN Journal Disclosure Form

As per ASN journal policy, I have disclosed any financial relationships or commitments I have held in the past 36 months as included below. I have listed my Current Employer below to indicate there is a relationship requiring disclosure. If no relationship exists, my Current Employer is not listed.

T. Ozrazgat-Baslanti reports the following:
Employer: University of Florida; and Patents or Royalties: University of Florida Research Foundation, Incorporated, Gainesville, FL (US).

I understand that the information above will be published within the journal article, if accepted, and that failure to comply and/or to accurately and completely report the potential financial conflicts of interest could lead to the following: 1) Prior to publication, article rejection, or 2) Post-publication, sanctions ranging from, but not limited to, issuing a correction, reporting the inaccurate information to the authors' institution, banning authors from submitting work to ASN journals for varying lengths of time, and/or retraction of the published work.

Name: Tezcan Ozrazgat-Baslanti
Manuscript ID: K360-2025-001097R2
Manuscript Title: Reinforcement learning for intraoperative hypotension management with consideration to postoperative acute kidney injury
Date of Completion: April 6, 2026
Disclosure Updated Date: April 6, 2026

ASN
American Society of Nephrology
JASN
Journal of the American Society of Nephrology
KIDNEY360
Accessing Our World From Every Angle
CJASN
Clinical Journal of the American Society of Nephrology

ASN Journal Disclosure Form

As per ASN journal policy, I have disclosed any financial relationships or commitments I have held in the past 36 months as included below. I have listed my Current Employer below to indicate there is a relationship requiring disclosure. If no relationship exists, my Current Employer is not listed.

P. Rashidi reports the following:
Employer: University of Florida; Andantech; MedBox Inc; Cognico Inc; Precision Circuits, Andantech; Consultancy: Precision Circuits; Ownership Interest: Andantech; MedBox Inc; Cognico Inc; Precision Circuits LLC; Research Funding: NIH/NSF; and Patents or Royalties: Pending patents filed by UF.

I understand that the information above will be published within the journal article, if accepted, and that failure to comply and/or to accurately and completely report the potential financial conflicts of interest could lead to the following: 1) Prior to publication, article rejection, or 2) Post-publication, sanctions ranging from, but not limited to, issuing a correction, reporting the inaccurate information to the authors' institution, banning authors from submitting work to ASN journals for varying lengths of time, and/or retraction of the published work.

Name: Parisa Rashidi
Manuscript ID: K360-2025-001097R3
Manuscript Title: Reinforcement learning for intraoperative hypotension management with consideration to postoperative acute kidney injury
Date of Completion: April 8, 2026
Disclosure Updated Date: April 8, 2026

ASN American Society of Nephrology | JASN Journal of the American Society of Nephrology | KIDNEY360 Accessing Our World From Every Angle | CJASN Clinical Journal of the American Society of Nephrology

ASN Journal Disclosure Form

As per ASN journal policy, I have disclosed any financial relationships or commitments I have held in the past 36 months as included below. I have listed my Current Employer below to indicate there is a relationship requiring disclosure. If no relationship exists, my Current Employer is not listed.

Y. Ren reports the following:
Employer: University of Florida

I understand that the information above will be published within the journal article, if accepted, and that failure to comply and/or to accurately and completely report the potential financial conflicts of interest could lead to the following: 1) Prior to publication, article rejection, or 2) Post-publication, sanctions ranging from, but not limited to, issuing a correction, reporting the inaccurate information to the authors' institution, banning authors from submitting work to ASN journals for varying lengths of time, and/or retraction of the published work.

Name: Yuanfang Ren
Manuscript ID: K360-2025-001097R1
Manuscript Title: Reinforcement learning for intraoperative hypotension management with consideration to postoperative acute kidney injury
Date of Completion: February 17, 2026
Disclosure Updated Date: August 11, 2025

ASN American Society of Nephrology | JASN Journal of the American Society of Nephrology | KIDNEY360 Accessing Our World From Every Angle | CJASN Clinical Journal of the American Society of Nephrology

ASN Journal Disclosure Form

As per ASN journal policy, I have disclosed any financial relationships or commitments I have held in the past 36 months as included below. I have listed my Current Employer below to indicate there is a relationship requiring disclosure. If no relationship exists, my Current Employer is not listed.

M. Ruppert reports the following:
Employer: University of Florida, Moss & Associates; and Ownership Interest: A,AAPL,ABT,AMD,AMN,AMZN,ASML,AVAV,AVGO,BAH,BAX,BRK-B,BROS,BSX,CACI,CAH,CAT,CDE,CI,COMP,COP,COST,CRWD,CVX,DAL,DASH,DGX,DKNG,DOCS,DRS,DVA,DXCM,EME,EQT,ERIC,EW,F,GEHC,GLW,GOOGL,GRMN,HAL,HAS,HCA,HL,HSIC,IBP,IQV,IRDM,JNJ,KBR,KGC,KLAR,KVUE,LDOS,LH,LHX,LLY,LMT,MCK,MDT,MELI,MRK,MRNA,MSFT,MTD,NEE,NEM,NKE,NOC,NOW,NVDA,OMCL,PANW,PD,PFE,PHG,PLTR,PSEC,RKLB,RMD,RTX,SBUX,SIRI,SLB,SMWB,STE,SUPN,SYK,T,TDG,TEM,TFC,TJX,TMO,TSM,TSLA,TTAN,UNH,UNP,VLO,VLTO,VST,VTEX,VVX,XOM.

I understand that the information above will be published within the journal article, if accepted, and that failure to comply and/or to accurately and completely report the potential financial conflicts of interest could lead to the following: 1) Prior to publication, article rejection, or 2) Post-publication, sanctions ranging from, but not limited to, issuing a correction, reporting the inaccurate information to the authors' institution, banning authors from submitting work to ASN journals for varying lengths of time, and/or retraction of the published work.

Name: Matthew M Ruppert
Manuscript ID: K360-2025-001097R3
Manuscript Title: Reinforcement learning for intraoperative hypotension management with consideration to postoperative acute kidney injury
Date of Completion: April 8, 2026
Disclosure Updated Date: April 8, 2026

ASN American Society of Nephrology
JASN Journal of the American Society of Nephrology
KIDNEY360 Accessing Our World From Every Angle
CJASN Clinical Journal of the American Society of Nephrology

ASN Journal Disclosure Form

As per ASN journal policy, I have disclosed any financial relationships or commitments I have held in the past 36 months as included below. I have listed my Current Employer below to indicate there is a relationship requiring disclosure. If no relationship exists, my Current Employer is not listed.

B. Shickel has nothing to disclose.

I understand that the information above will be published within the journal article, if accepted, and that failure to comply and/or to accurately and completely report the potential financial conflicts of interest could lead to the following: 1) Prior to publication, article rejection, or 2) Post-publication, sanctions ranging from, but not limited to, issuing a correction, reporting the inaccurate information to the authors' institution, banning authors from submitting work to ASN journals for varying lengths of time, and/or retraction of the published work.

Name: Benjamin Shickel
Manuscript ID: K360-2025-001097R1
Manuscript Title: Reinforcement learning for intraoperative hypotension management with consideration to postoperative acute kidney injury
Date of Completion: February 18, 2026
Disclosure Updated Date: August 12, 2025

ASN American Society of Nephrology | JASN Journal of the American Society of Nephrology | KIDNEY360 Accessing Our World From Every Angle | CJASN Clinical Journal of the American Society of Nephrology

ASN Journal Disclosure Form

As per ASN journal policy, I have disclosed any financial relationships or commitments I have held in the past 36 months as included below. I have listed my Current Employer below to indicate there is a relationship requiring disclosure. If no relationship exists, my Current Employer is not listed.

K. Singh reports the following:
Employer: University of California, San Diego; Consultancy: Google; and Advisory or Leadership Role: Google (Consumer Health Advisory Panel).

I understand that the information above will be published within the journal article, if accepted, and that failure to comply and/or to accurately and completely report the potential financial conflicts of interest could lead to the following: 1) Prior to publication, article rejection, or 2) Post-publication, sanctions ranging from, but not limited to, issuing a correction, reporting the inaccurate information to the authors' institution, banning authors from submitting work to ASN journals for varying lengths of time, and/or retraction of the published work.

Name: Karandeep Singh
Manuscript ID: K360-2025-001097R1
Manuscript Title: Reinforcement learning for intraoperative hypotension management with consideration to postoperative acute kidney injury
Date of Completion: February 17, 2026
Disclosure Updated Date: October 6, 2025
