# Supplementary material for: Reinforcement Learning for Intraoperative Hypotension Management with Consideration to Postoperative Acute Kidney Injury
Source: Kidney360. 2026 May 5;7(7):1535–44. doi: 10.34067/KID.0000001212 (PMC13450969; doi:10.34067/KID.0000001212)
Supplement: Supplementary file 2 [file kidney360-7-1535-s002.pdf]

Supplemental Material

Reinforcement learning for intraoperative hypotension management with consideration to postoperative acute kidney injury

Esra Adiyeke, Tianqi Liu[*], Venkata Sai Dheeraj Naganaboina[*], Han Li, Tyler J. Loftus, Yuanfang Ren, Benjamin Shickel, Matthew M. Ruppert, Karandeep Singh, Ruogu Fang, Parisa Rashidi, Azra Bihorac[#], Tezcan Ozrazgat-Baslanti[#]

* These authors have contributed equally

# These authors have contributed equally as senior authors

This supplemental material has been provided by the authors to give readers additional information about their work.

Supplemental Material Contents

Supplemental Methods

Supplemental Figure 1. Figure illustrating derivation of the study population.

Supplemental Figure 2. Workflow pipeline.

Supplemental Figure 3. The silhouette score (A) and within-cluster sum of squares (B) as functions of the number of clusters.

Supplemental Figure 4. Comparison of actions proposed by the trained agent (A) and taken by physician agent (B), average return per surgery (C) and the relationship between the return of physicians' treatments and postoperative AKI within the first 3 days following surgery in test set (D), difference between given and recommended dose over all 15-minute intervals for IV fluids (E) and for vasopressors (F) per surgery.

Supplemental Figure 5. Comparison between doses suggested by RL model and physician's administration for a surgical session without postoperative AKI within the first 3 days after surgery. (Example surgery 1)

Supplemental Figure 6. Comparison between doses suggested by RL model and physician's administration for a surgical session without postoperative AKI within the first 3 days after surgery. (Example surgery 2)

Supplemental Figure 7. Comparison between doses suggested by RL model and physician's administration for a surgical session with postoperative AKI within the first 3 days after surgery. (Example surgery 3)

Supplemental Figure 8. Comparison between doses suggested by RL model and physician's administration for a surgical session with postoperative AKI within the first 3 days after surgery. (Example surgery 4)

Supplemental Table 1. Table listing characteristics of input variables.

Supplemental Table 2. Detailed cohort characteristics.

Supplemental Table 3. Norepinephrine conversion factors.

Supplemental Table 4. Intravenous fluid discretization

Supplemental Table 5. Vasopressor discretization

Supplemental Table 6. Hypotension and AKI outcomes.

Supplemental Methods

A. Cohort

Data from the full patient cohort were divided into a development cohort, comprising patients admitted between June 1, 2014 and November 29, 2018, and a validation cohort, from November 30, 2018 to September 20, 2020 in a 70% to 30% ratio. Patients were excluded if they had any of the following cases: 1) end stage kidney disease present, 2) age < 18 on admission, 3) not an inpatient encounter, 4) missing admission or discharge date or missing surgery stop time, 4) surgery performed for organ donation, a minor gastrointestinal procedure, or a minor pain management procedure, anesthesia administered outside of the operating room, 5) the surgery was < 60 minutes, 6) 3-day or 7-day acute kidney injury (AKI) status was missing due to insufficient serum creatinine data available, 7) cardiac surgeries. (Supplemental Figure 1).

B. Dosage pre-processing and action space

Intravenous fluids (IV) included boluses and continuous infusions of crystalloid and colloid solutions. Vasopressors included dopamine, epinephrine, norepinephrine, phenylephrine, and vasopressin. Outliers were capped to expert defined clinically plausible values identified utilizing maximum doses used in refractory shock (Supplemental Table 3). Vasopressor dosages were converted into norepinephrine-equivalents using dosage conversions as listed in Supplemental Table 3.

Using norepinephrine-equivalent dosages normalized to rate and weight, we developed a discrete 5 x 5 action space of vasopressors and IV fluid dosages given in 15-minute intervals. Thresholds for action bins were chosen by selecting percentile thresholds for points of high rate of dosage increase and were validated through expert review. We converted each drug dosage at every timestep into an integer representing its bin. No drug dosage was encoded as bin 1, the lowest dosage category was encoded as bin 2 and the highest dosage category was encoded as bin 5. Thus, interventions were represented as tuples of total IV and vasopressor dosages per 15-minute interval.

C. Model Details

Surgery can be conceptualized as a sequential decision-making process, and to train an agent, we used the Reinforcement Learning Algorithm with 17 variables; that consists of 16 clinical variables (age, sex, Charlson comorbidities index, body mass index, heart rate, systolic blood pressure, mean arterial pressure, body temperature, respiratory rate, minimum alveolar concentration, SF ratio, peak inspiratory pressure, end-tidal carbon dioxide, average mean arterial blood pressure of past 48 hours, cumulative IV and cumulative pressor administered during surgery) and cluster index. The physiological state of a patient was represented by these selected variables, and thus the surgical decision-making process could be formulated as a partially observable Markov Decision Process (MDP). The model free MDP was defined by a tuple $\{S, A, R, \gamma\}$ with:

1. S is the state of a patient (patient health states)
2. A is the finite set of actions for state S (in our model, the doses intravenous fluids and vasopressors are discretized into 25 actions)
3. $R(s')$ is the immediate reward received for transitioning to next state S'.
4. Γ is the discounting factor, that indicates the decay of influences for future rewards than an immediate reward.

Our model operated on 15-minute time intervals, within which multiple measurements were recorded. We considered resampling by averaging all measurements within each interval as a preprocessing step before inputting the data into the model. We set Γ to 0.99 in the model.

We utilized the K-means++ clustering algorithm to categorize the resampled data points into distinct clusters. Specifically, we used a total of 16 clinical features derived from the patient data for clustering, to provide a comprehensive representation of the patient's condition. The K-means++ algorithm was chosen for its efficiency in selecting initial cluster centroids. Cluster membership was assigned based on the closest centroids, ensuring that each data point was grouped with the most similar patient states. We determined an optimal number of 200 clusters using the elbow method for silhouette analysis. We developed 50 distinct RL models for 50 different clustering outcomes obtained by different random initializations to account for the variability in policy values. This discretization allows us to group patients with similar medical conditions and tailor specific treatment strategies for each group. Additionally, the cluster number was included as a feature in the overall feature set before training, providing our model with an additional layer of insight into patient state transitions.

Reward Function

We developed a reward function as a combination of long- and short-term rewards with two major parts to consider. The reward function is the sum of rewards of two parts:

$$r = r_{aki} + r_{hypo} \quad \text{Eq. 1}$$

Part 1: Postoperative AKI outcome: This acts as the long-term reward/penalty utilizing a dedicated reward of +15 if no AKI occurred within 3 days following surgery, and -15 for if AKI occurred within 3 days following surgery.

$$r_{aki} = \begin{cases} 15, & no\ AKI\ within\ 3\ days\ of\ surgery \\ -15, & AKI\ within\ 3\ days\ of\ surgery \end{cases} \quad \text{Eq. 2}$$

Part 2: Hypotension: The function adds a penalty of 1.75 if the state is hypotensive, that is, if mean arterial pressure (MAP) is less than 65 mmHg or 20% lower than baseline MAP. The baseline MAP was calculated by taking the median non-invasive MAP within the 48 hours prior to the first surgery start date-time that was between 60 and 110 mmHg.

$$r_{hypo} = \begin{cases} -1.75, & MAP_t < 65mmHg\ or\ MAP_t < 0.8 \times MAP_{baseline} \\ 0, & otherwise \end{cases} \quad \text{Eq. 3}$$

Evaluation of physicians' action:

We used Dueling Double Deep Q Networks (D3QN) as architecture for the RL model to learn an optimal policy (agent policy) and test it. We performed an evaluation of the real actions (the policy) of physicians using temporal difference (TD) for the Q function learning with observed states, actions and rewards tuples in surgeries. The Q function learning is computed iteratively by the following formula:

$$Q^{\pi}(s,a) \leftarrow Q^{\pi}(s,a) + \alpha\,(r + \gamma Q^{\pi}(s^{'}, a^{'}) - Q^{\pi}(s,a)) \quad \text{Eq. 4}$$

where the α is the learning rate and r is the immediate reward.

The loss function of the D3QN is denoted as:

$$L_{DQN} = \left(\left(r + \gamma \max_{a_{t+1}} Q(s_{t+1}, a_{t+1},; \theta^{target})\right) - Q\left(s, a; \theta^{pred}\right)\right)^2 \quad \text{Eq. 5}$$

The training experiences are recorded from physicians, therefore the action recommended by AI agent should have similar distribution with actions from physicians to ensure the safety of the agent policy. We added a KL divergence penalty to the loss function to constrain the action distribution for AI agent from diverging from physicians' actions:

$$L = L_{DQN} + \alpha L_{KL} \quad \text{Eq. 6}$$

$$L_{KL} = D_{KL}(D_{Physician} \| D_{Agent}) = \sum_{i=1}^{N} [p_{phy}(x_i) log p_{phy}(x_i) - p_{phy}(x_i) log q_{agent}(x_i)] \quad \text{Eq. 7}$$

Estimation of the AI policy

Our model learned an optimal policy, as we refer as agent policy, in theory with the goal of maximizing the sum of rewards to avoid long-term AKI and short-term hypotension. The agent policy will start with a random policy that was iteratively evaluated and then improved until converging to an optimal solution. After convergence, the agent policy π^* corresponded to actions with the highest state-action value:

$$\pi^*(s) \leftarrow argmax_a Q^{\pi^*}(s,a),\ \forall s \qquad \text{Eq. 8}$$

D. Model Evaluation

Weighted Importance Sampling (WIS)

Traditional reinforcement learning applications have typically been tested in simulated environments where the evaluation of performance of reinforcement learning algorithms can be easily quantified, such as in video games. However, unlike synthetic environments, clinical settings do not permit real-time experimentation with unvalidated policies. Off-policy evaluation allows for retrospective assessment of a target policy (e.g., model suggested treatment strategy) under a different behavior policy (e.g., physician decision making). Consequently, off-policy evaluation is adopted for our task to evaluate our trained AI agent (π_e) based on physicians' policies. Weighted Importance Sampling (WIS) takes the idea to reweight the rewards in the historical data (physicians' policy π_b) by the importance sampling ratio between π_e and π_b.

The per-step importance ratio is defined at step *t* as:

$$\rho_t = \frac{\pi_e(a_t|s_t)}{\pi_b(a_t|s_t)} \qquad \text{Eq. 9}$$

The cumulative importance ratio up to step t is formulated as:

$$\rho_{1:t} = \prod_{t'=1}^{t} \rho_{t'} \qquad \text{Eq. 10}$$

where t is the end of a surgery. However, the cumulative importance ratio is not stable for long surgeries due to the large number of steps; that is 15-min intervals. When most ρ_t are larger than 1, the product value will soon exponentially explode to a very large number, which makes the evaluation not stable. To solve this problem, we proposed a new cumulative ratio as follows:

$$\rho'_{1:t} = ln\rho_{1:t} = ln\prod_{t'=1}^{t} \rho_{t'} \qquad \text{Eq. 11}$$

The ln can compress the range of input value. But if the $\rho_{1:t}$ is smaller than 1, the output of ln function is smaller than 0. Since a negative cumulative ratio is not meaningful in this context, we instead used the Softmax function, which maps values from the range $(-\infty,\infty)$ to $[0,1]$.

$$\rho''_{1:t} = \frac{1}{1+e^{-\rho'_{1:t}}} = \frac{1}{1+e^{-ln\rho_{1:t}}} = \frac{1}{1+\frac{1}{\rho_{1:t}}} \qquad \text{Eq. 12}$$

We then calculated the average cumulative importance ratio for a dataset D with $|D|$ number of trajectories, that is the number of surgeries in this case:

$$w_D = \frac{\sum_{i=1}^{|D|} \rho''^{(i)}_{1:t_i}}{|D|} \qquad \text{Eq. 13}$$

The trajectory-wise WIS estimator is given by:

$$V_{WIS}^{(i)} = \frac{\rho''^{(i)}_{1:t^i}}{w_D}\left(\sum_{t=1}^{t^i} \gamma^{t-1} r_t\right) \quad \text{Eq. 14}$$

Then the WIS estimator was calculated as the average estimate over all trajectories:

$$\text{WIS} = \frac{1}{|D|}\sum_{i=1}^{|D|} V_{WIS}^{(i)} \quad \text{Eq. 15}$$

where $V_{WIS}^{(i)}$ is WIS applied to the i-th trajectory.

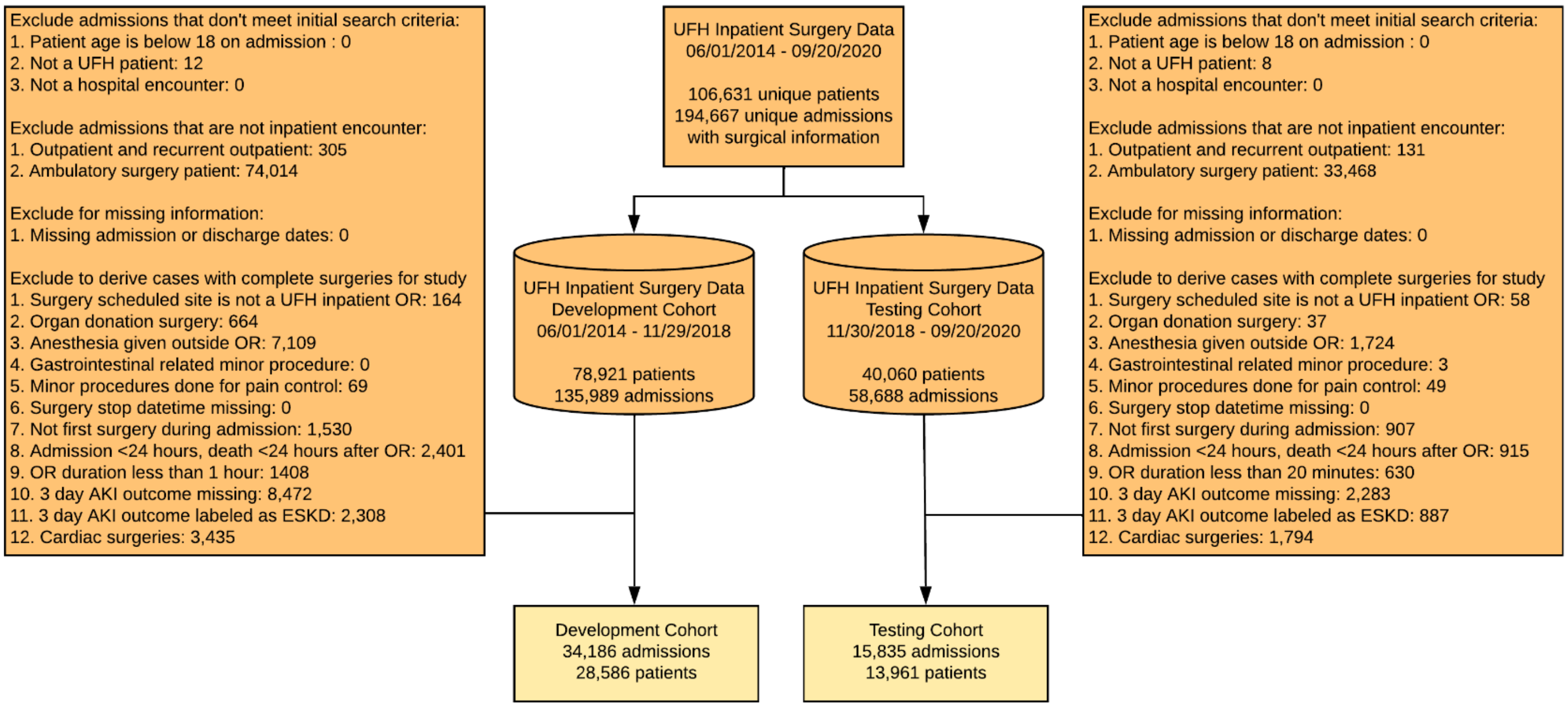

Supplemental Figure 1. Figure illustrating derivation of the study population

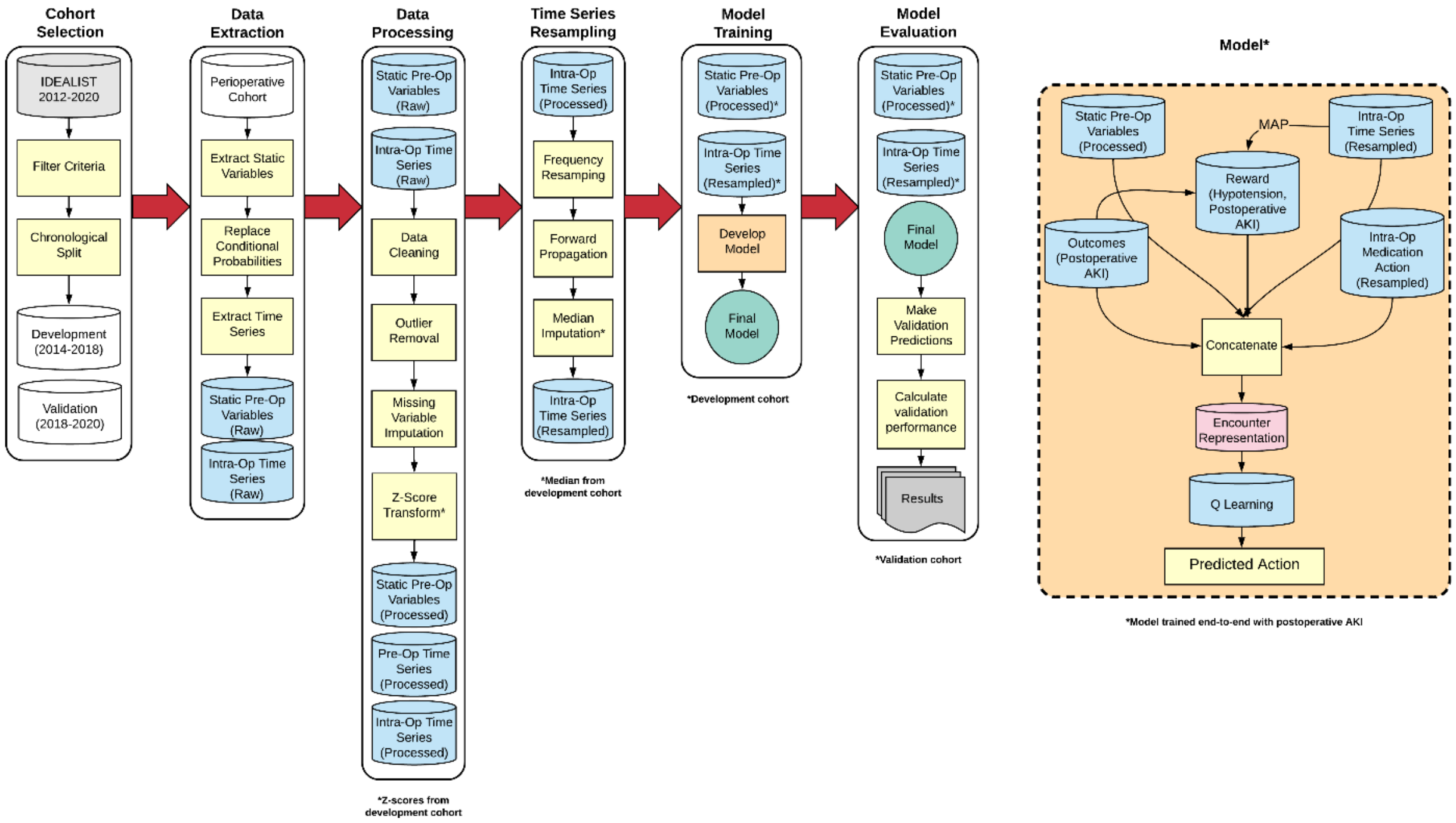

Supplemental Figure 2. Workflow pipeline. (Abbreviations: AKI, acute kidney injury)

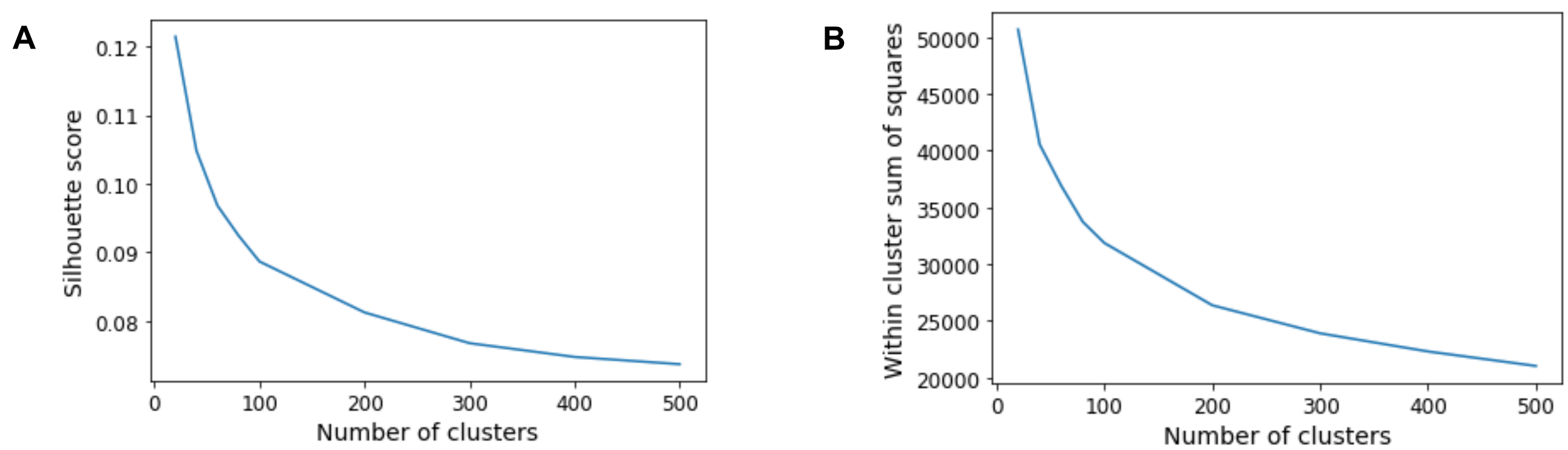

Supplemental Figure 3. The silhouette score (A) and within-cluster sum of squares (B) as functions of the number of clusters.

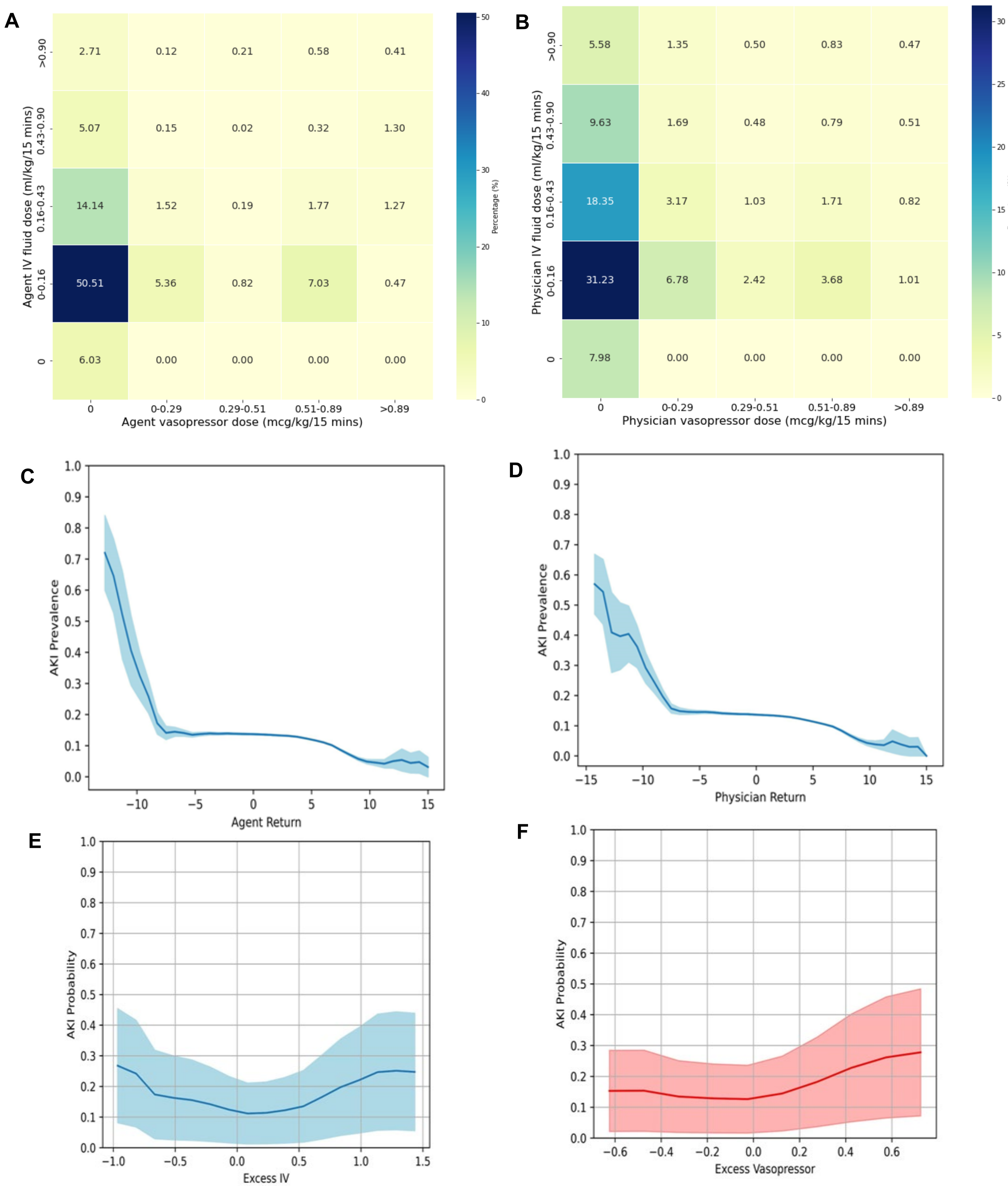

Supplemental Figure 4. Comparison of actions proposed by the trained agent (A) and taken by physician agent (B), average return per surgery (C) and the relationship between the return of physicians' treatments and postoperative AKI within the first 3 days following surgery in test set (D), difference between given and recommended dose over all 15-minute intervals for IV fluids (E) and for vasopressors (F) per surgery.

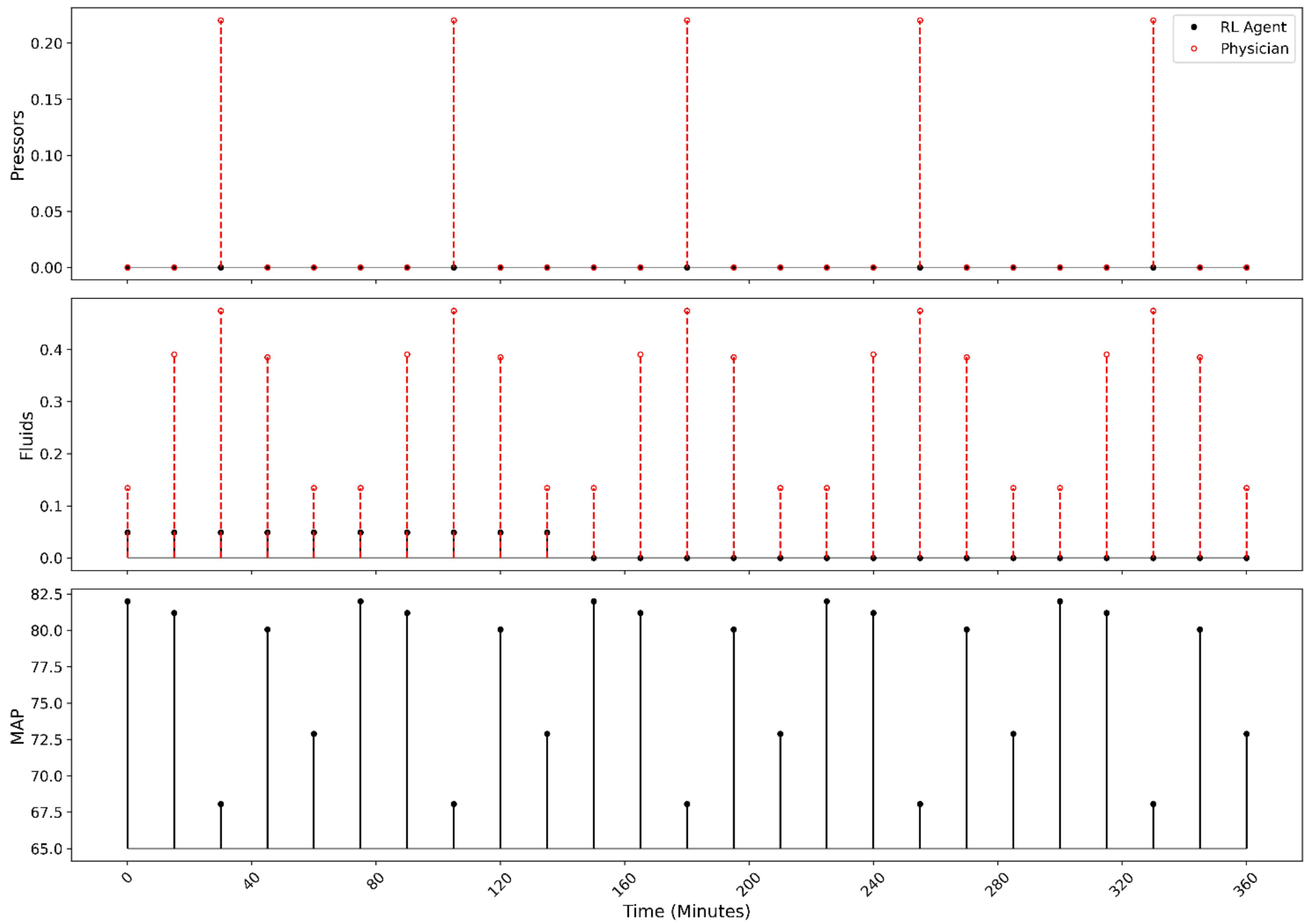

Supplemental Figure 5. Comparison between doses suggested by RL model and physician's administration for a surgical session without postoperative AKI within the first 3 days after surgery. (Example surgery 1)

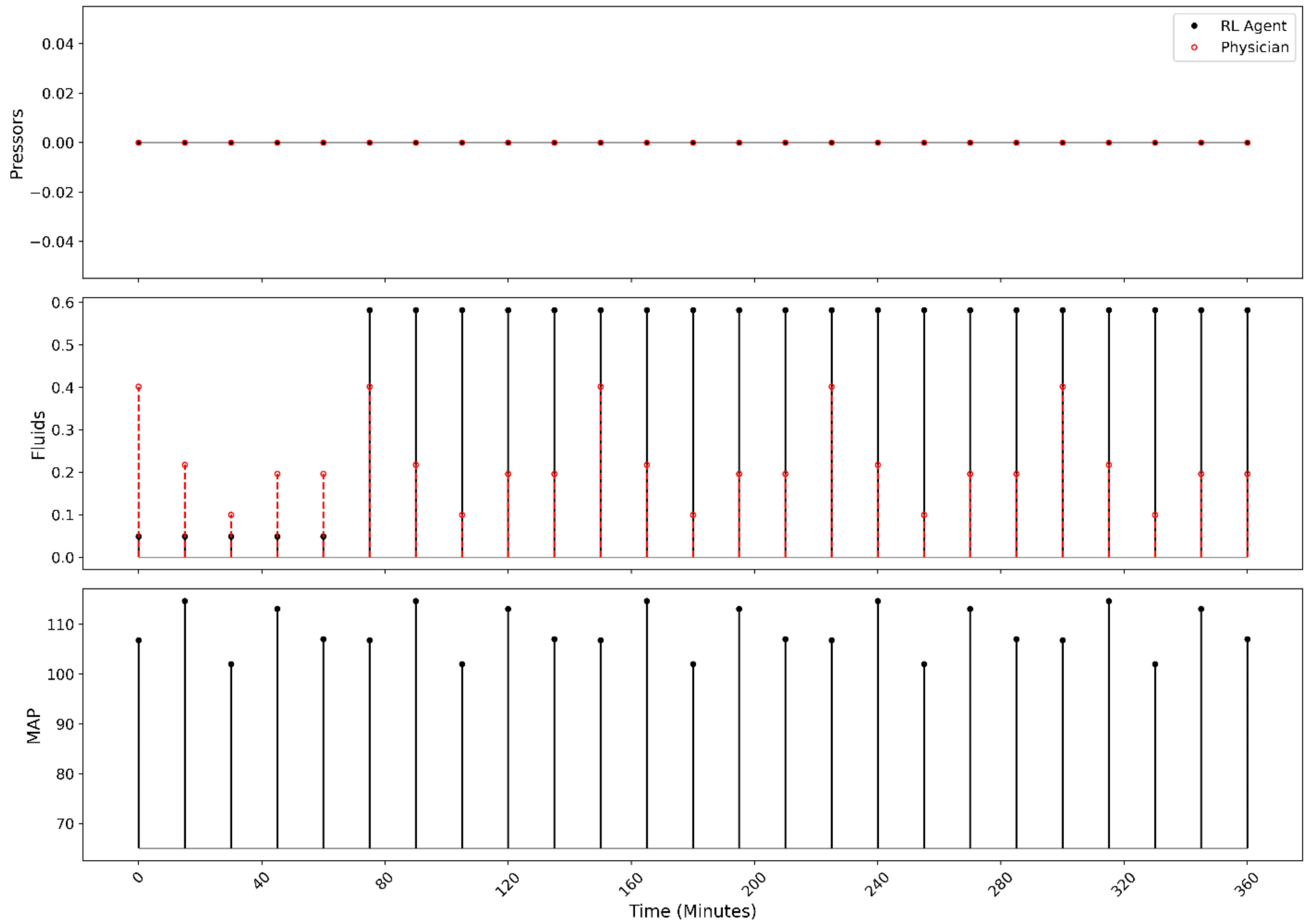

Supplemental Figure 6. Comparison between doses suggested by RL model and physician's administration for a surgical session without postoperative AKI within the first 3 days after surgery. (Example surgery 2)

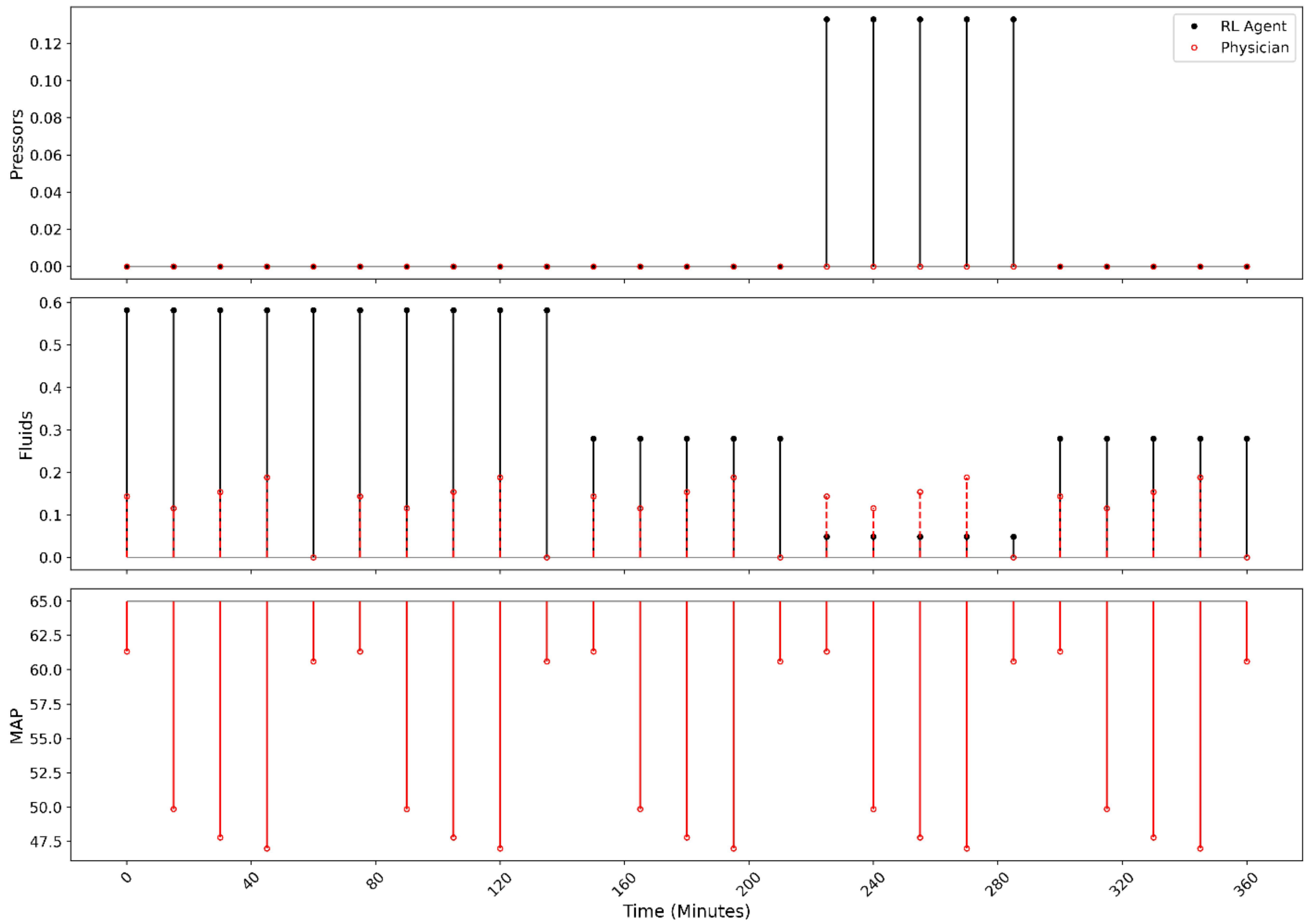

Supplemental Figure 7. Comparison between doses suggested by RL model and physician's administration for a surgical session with postoperative AKI within the first 3 days after surgery. (Example surgery 3)

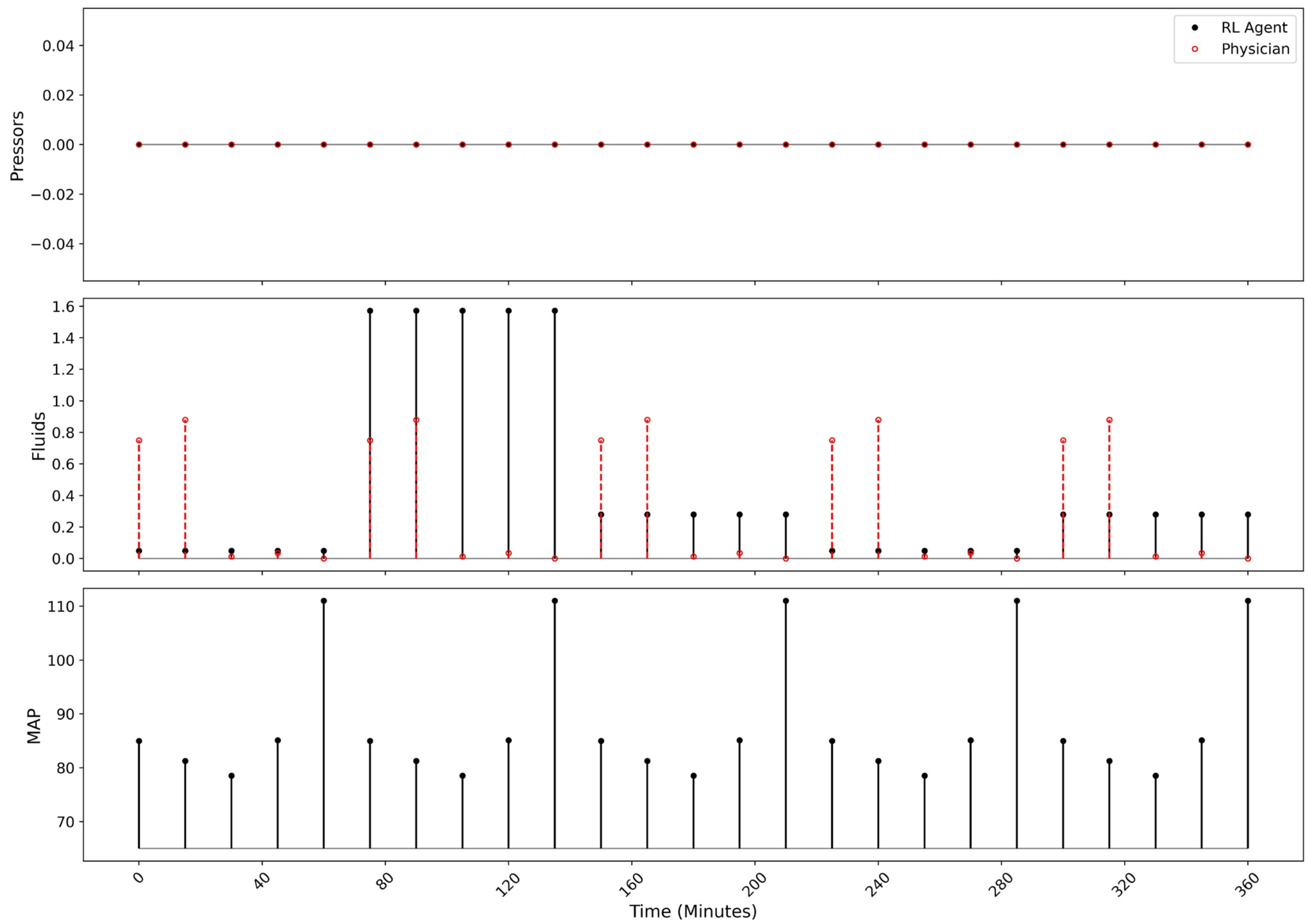

Supplemental Figure 8. Comparison between doses suggested by RL model and physician's administration for a surgical session with postoperative AKI within the first 3 days after surgery. (Example surgery 4)

Supplemental Table 1. Table listing characteristics of input variables.

Variable	Type of Variable	Data Source	Number of Categories	Type of Preprocessing
**Demographic Variables**				
Age (years)	Continuous	Derived		Imputation of outliers[a]
Sex	Binary	Raw	2	
Body Mass Index	Continuous	Raw		Imputation of outliers[a]
**Comorbidities**				
Charlson's Comorbidity Index	Nominal	Derived	18	Categorization
**Intraoperative Medications[b]**				
Vasopressors	Continuous	Raw		Data cleaning[c]
Intravenous (IV) fluid	Continuous	Raw		Data cleaning[c]
**Physiological Intraoperative Time Series**				
Systolic blood pressure, mmHg	Continuous	Raw		Data cleaning[c]
Mean Arterial Pressure, mmHg	Continuous	Raw		Data cleaning[c]
Minimum alveolar concentration	Continuous	Raw		Data cleaning[c]
Heart rate, bpm	Continuous	Raw		Data cleaning[c]
Temperature (°C)	Continuous	Raw		Data cleaning[c]
End-tidal CO2 (EtCO2)	Continuous	Raw		Data cleaning[c]
Peak Inspiratory Pressure (PIP)	Continuous	Raw		Data cleaning[c]
Respiratory Rate	Continuous	Raw		Data cleaning[c]
Oxygen saturation to Fraction of inspired oxygen ratio (SPO2/FIO2 ratio)	Continuous	Derived		Data cleaning[c]

A different set of variables was kept in final models (preoperative or intraoperative) from the input set provided in the table.

[a] For continuous variables, observations that fell in the top and bottom 1% of the distribution were considered as outliers and imputed by neighborhood values (i.e., above 99%) are imputed randomly from a uniform distribution defined over [95%, 99.5%] percentiles and below 1% are imputed randomly from another uniform distribution defined over [0.5%, 5%] percentiles.

[b] Medications were taken during surgery using RxNorms data grouped into drug classes according to the US, Department of Veterans Affairs National Drug File-Reference Terminology.

[c] We used observations for the first surgery, in case multiple surgeries exist. We averaged values if multiple observations exist at a time point.

Supplemental Table 2. Detailed cohort characteristics.

Features	Development Cohort	Test Cohort
Number of encounters, n	34,186	15,835
**Demographic information**		
Age, years, mean (SD)	57 (17)	59 (17)
Sex, n (%)		
Male, n (%)	17,031 (50)	7,909 (50)
Female, n (%)	17,155 (50)	7,926 (50)
Race, n (%)		
African American	4,776 (14)	2,233 (14)
Other[a]	2,164 (6)	972 (6)
White	26,743 (78)	12,360 (78)
Missing	503 (1)	270 (2)
Ethnicity, n (%)		
Hispanic	1530 (4)	756 (5)
Non-Hispanic	32,063 (94)	14,711 (93)
Body Mass Index, median (IQR)	28 (24, 34)	28 (24, 34)
**Comorbidities, n (%)**		
Charlson comorbidity index, median (IQR)	4 (2, 6)	4 (2, 6)
Alcohol or drug abuse	5,353 (16)	2,587 (16)
Myocardial Infarction	2,384 (7)	1,156 (7)
Congestive Heart Failure	4,653 (14)	2,488 (16)
Peripheral Vascular Disease	6,967 (20)	3,644 (23)
Cerebrovascular Disease	5,642 (17)	2,854 (18)
Chronic Pulmonary Disease	1,0516 (31)	5,333 (34)
Cancer	1,0186 (30)	4,465 (28)
Metastatic Carcinoma	3,451 (10)	1,678 (11)
Liver Disease	5,232 (15)	2,549 (16)
Diabetes	8,252 (24)	3,916 (25)
Hypertension	21,843 (64)	10,642 (67)
Obesity	11,393 (33)	7,070 (45)
Fluid and electrolyte disorders	10,301 (30)	6,120 (39)
Valvular Disease	3,561 (10)	2,048 (13)
Coagulopathy	4,674 (14)	2,173 (14)
Weight Loss	5,212 (15)	2,589 (16)
Depression	9,544 (28)	4,805 (30)
Chronic Anemia	6,792 (20)	4,183 (26)
Chronic Kidney Disease	5,718 (17)	3,019 (19)
Reference estimated glomerular filtration rate (mL/min/1.73 m^2), median (IQR)	95 (82, 110)	93 (81, 108)
Baseline mean arterial pressure mmHg, median (IQR)[b]	86 (78, 94)	87 (79, 95)

Features	Development Cohort	Test Cohort
**Surgical information, n (%)**		
Time from Admission to Surgery, days, median (IQR)	3 (2, 22)	3 (2, 23)
Emergency admission	12,243 (36)	5,763 (36)
Admission type		
Surgery	13,080 (38)	6,521 (41)
Medicine	16,796 (49)	6,920 (44)
Transferred from another hospital	5,666 (17)	2,667 (17)
Anesthesia Type		
General	31,281 (92)	14,941 (94)
Local/regional	2,905 (8)	894 (6)
Surgery Type		
Orthopedic Surgery	10,109 (30)	4,341 (27)
Neurosurgery	3,744 (11)	2,546 (16)
Vascular Surgery	3,420 (10)	1,704 (11)
Other	3,234 (9)	2,029 (13)
Urology	3,768 (11)	1,231 (8)
Gastrointestinal Surgery	2,798 (8)	1,165 (7)
Ear Nose Throat	1,873 (5)	855 (5)
OB Gynecology	1,291 (4)	541 (3)
Surgical Oncology	1,495 (4)	449 (3)
Plastic Surgery	619 (2)	277 (2)
Transplantation	575 (2)	170 (1)
Burn Surgery	883 (3)	321 (2)
Pediatric Surgery	270 (1)	122 (1)
Ophthalmology	95 (0)	79 (0)
Medicine Gastroenterology	12 (0)	5 (0)
**Intraoperative vitals**		
Systolic blood pressure		
Measured value, mmHg, median IQR	114 (102 , 130)	116.0 (104 , 132)
Total measurements, n, IQR	73 (47, 149)	81.0 (50, 179)
Encounters missing, n (%)	21 (0.1)	11 (0.1)
Mean arterial pressure		
Measured value, mmHg, median IQR	79 (70, 90)	82.0 (72, 93)
Total measurements, n, IQR	73 (47, 150)	81.0 (50, 179)
Encounters missing, n (%)	21 (0.1)	11 (0.1)
Heart rate		
Measured value, bpm, median IQR	75 (65.5, 86.5)	75.0 (66., 86.5)
Total measurements, n, IQR	175 (121.8, 257)	179.0 (124, 259)
Encounters missing, n (%)	22 (0.1)	9 (0.1)
Oxygen saturation (SpO2)		
Measured value, %, median IQR	99.1 (97.5, 100)	99 (97.2, 100)
Total measurements, n, IQR	177 (120, 268)	184.0 (123, 277)
Encounters missing, n (%)	43 (0.1)	18 (0.1)
Fraction of inspired oxygen (FiO2)		
Measured value, %, median IQR	40 (40, 40)	40 (40, 40)
Total measurements, n, IQR	187 (130, 280)	195.0 (135, 291)
Encounters missing, n (%)	19 (0.1)	8 (0.1)
End-tidal carbon dioxide (EtCO2)		
Measured value, mmHg, median IQR	34 (32, 37)	35 (33, 38)
Total measurements, n, IQR	149 (82, 238)	2 (0, 163)
Encounters missing, n (%)	3,548 (10.4)	7,254 (45.8)

Features	Development Cohort	Test Cohort
Respiration rate		
Measured value, breaths/minute, median IQR	10 (8, 12)	12 (10, 14)
Total measurements, n, IQR	183 (125, 275)	124 (3, 214)
Encounters missing, n (%)	75 (0.2)	782 (4.9)
Peak inspiratory pressure		
Measured value, mmHg, median IQR	18 (14, 23)	18 (14, 22)
Total measurements, n, IQR	183 (126, 276)	176 (109, 271)
Encounters missing, n (%)	396 (1.2)	1,567 (9.9)
Minimum alveolar concentration		
Measured value, median IQR	0.6 (0.4, 0.8)	0.6 (0.3, 0.8)
Total measurements, n, IQR	160 (102, 242)	173 (118, 252)
Encounters missing, n (%)	3,234 (9.5)	1,449 (9.2)
Core temperature		
Measured value, degrees Celsius, median IQR	36.8 (36.3, 37.3)	36.9 (36.3, 37.4)
Total measurements, n, IQR	126 (65, 202)	106 (13, 184)
Encounters missing, n (%)	1,811 (5.3)	1,233 (7.8)
**Intraoperative medications, median (IQR)[c]**		
Vasopressor total dose per 15 min (mcg/kg)	0 (0, 0.1)	0 (0, 0.1)
Intravenous fluids total dose per 15 min (ml/kg)	0.1 (0, 0.4)	0.1 (0, 0.4)
**Intraoperative hypotension, n (%)**	24,144 (70)	9,677 (61)
**Complications, n (%)**		
Acute kidney injury	5,649 (17)	27,72 (18)
Acute kidney injury within 3 days after surgery	3,870 (11)	1,856 (12)
Acute kidney injury within 7 days after surgery	4,637 (14)	2,242 (14)
Cardiovascular complication	4,509 (13)	2,508 (16)
Neurological complication and delirium	4,167 (12)	2,615 (17)
Prolonged ICU stay	8,533 (25)	4,694 (30)
Prolonged mechanical ventilation	2,155 (6)	941 (6)
Sepsis	2,891 (8)	1,491 (9)
Venous thromboembolism	1,592 (5)	858 (5)
Wound complication	5,389 (16)	3,540 (22)
30-day mortality	749 (2)	320 (2)
90-day mortality	1,350 (4)	475 (3)

Abbreviations. SD, standard deviation; IQR, interquartile range; ICU, Intensive Care Unit.

[a] Other race includes American Indian/Alaskan Native, Asian, Native Hawaiian/other Pacific Islander, and multiracial.
[b] Baseline MAP was calculated as the median non-invasive MAP within 48 hours prior to the start of the first surgery, limited to values between 60 and 110 mmHg.

[c] Values were calculated for 15-minute resampled series.

Supplemental Table 3. Maximum allowed dose for outlier cleaning and conversion factors.

**Vasopressor**	**Maximum Allowed Dose**	**Norepinephrine Equivalent Dose***
Norepinephrine	3.3 mcg/kg/min	1
Epinephrine	2 mcg/kg/min	1
Vasopressin	0.1 units/min	2.5
Phenylephrine	10 mcg/kg/min	0.1
Dopamine	50 mcg/kg/min	0.01

*Approximate conversion of vasopressin dose in units/min to equivalent norepinephrine dose in mcg/kg/min, normalized to 100 kg body weight.

Norepinephrine equivalent dose (mcg/kg/min) = dose (mcg/kg/min)*coefficient, for epinephrine, phenylephrine, and dopamine

Norepinephrine equivalent dose (mcg/kg/min) = dose (units/min)*coefficient*(weight in kg/100kg) for vasopressin

Supplemental Table 4. Intravenous (IV) fluid dose discretization.

**IV fluid Action**	**1**	**2**	**3**	**4**	**5**
Range of dose	0 (None)	0<x<=0.16 ml/kg/15 mins (0-50%)	0.16<x<=0.43 ml/kg/15 mins (50-75%)	0.43 < x<=0.90 ml/kg/15 mins (75-90%)	x>0.90 ml/kg/15 mins (90-100%)

Supplemental Table 5. Vasopressor dose discretization.

**Pressor Action**	**1**	**2**	**3**	**4**	**5**
Range of dose	0 (None)	0<x<=0.29 mcg/kg/15 mins (0-50%)	0.29<x<=0.51 mcg/kg/15 mins (50-75%)	0.51 < x<=0.89 mcg/kg/15 mins (75-90%)	x>0.89 mcg/kg/15 mins (90-100%)

Supplemental Table 6. Intraoperative hypotension and postoperative acute kidney injury (p value<0.001).

	**Postoperative Acute Kidney Injury**	
**Intraoperative Hypotension, n (%)**	No	Yes
No	14,519 (90)	1,681 (10)
Yes	29,776 (88)	4,045 (12)
